# Historical exposure to chemicals reduces tolerance to novel chemical stress in *Daphnia* (waterflea)

**DOI:** 10.1111/mec.16451

**Published:** 2022-04-15

**Authors:** Muhammad Abdullahi, Jiarui Zhou, Vignesh Dandhapani, Anurag Chaturvedi, Luisa Orsini

**Affiliations:** ^1^ Environmental Genomics Group School of Biosciences the University of Birmingham Birmingham UK; ^2^ The Alan Turing Institute British Library London UK

## Abstract

Until the last few decades, anthropogenic chemicals used in most production processes have not been comprehensively assessed for their risk and impact on wildlife and humans. They are transported globally and usually end up in the environment as unintentional pollutants, causing long‐term adverse effects. Modern toxicology practices typically use acute toxicity tests of unrealistic concentrations of chemicals to determine their safe use, missing pathological effects arising from long‐term exposures to environmentally relevant concentrations. Here, we study the transgenerational effect of environmentally relevant concentrations of five chemicals on the priority list of international regulatory frameworks on the keystone species *Daphnia magna*. We expose *Daphnia* genotypes resurrected from the sedimentary archive of a lake with a known history of chemical pollution to the five chemicals to understand how historical exposure to chemicals influences adaptive responses to novel chemical stress. We measure within‐ and transgenerational plasticity in fitness‐linked life history traits following exposure of “experienced” and “naive” genotypes to novel chemical stress. As the revived *Daphnia* originate from the same genetic pool sampled at different times in the past, we are able to quantify the long‐term evolutionary impact of chemical pollution by studying genome‐wide diversity and identifying functional pathways affected by historical chemical stress. Our results suggest that historical exposure to chemical stress causes reduced genome‐wide diversity, leading to lower cross‐generational tolerance to novel chemical stress. Lower tolerance is underpinned by reduced gene diversity at detoxification, catabolism and endocrine genes in experienced genotypes. We show that these genes sit within pathways that are conserved and potential chemical targets in other species, including humans.

## 1 INTRODUCTION

Anthropogenic chemicals used in most production processes are transported globally, and usually end up in the environment as unintentional pollutants that harm humans and damage the environment (Wang et al., 2020). Until the last few decades, there were no comprehensive assessments of the risk and impact of anthropogenic chemicals on wildlife and humans (Dulio et al., 2018) and premarket toxicity has not been evaluated (Brooks et al., 2020).

Although toxicology has modernized significantly (Choudhuri et al., 2018), predictive frameworks rely on acute toxicity estimates that are limited in scope and do not necessarily reflect realistic exposure scenarios *in situ* (Kanno, 2016). These approaches also do not account for the cumulative toxicity that may arise from long‐term exposures to sublethal concentrations of a chemical or chemical mixtures (Blair et al., 2015). Long‐term exposure to environmentally relevant concentrations of anthropogenic chemicals has been shown to cause loss of genetic diversity, with consequences for adaptive potential to novel stress (Bijlsma & Loeschcke, 2012; Fasola et al., 2015; Ribeiro et al., 2012). Chemical stress has documented adverse effects on wildlife's ecological endpoints, including developmental defects and survival (Blahova et al., 2020; Jantzen et al., 2016), behaviour and metabolism (Xia et al., 2015), delayed growth and metamorphosis (Yoon et al., 2019), embryonic development (Balbi et al., 2018), fecundity and sexual maturity (Liu et al., 2017).

In an effort to understand the impact of long‐term chemical stress on *Daphnia* fitness and susceptibility to novel chemical stress, here we study the transgenerational effects of five chemicals on the priority list of international regulatory frameworks because of their widespread presence in the environment and their potential adverse effects (Chang et al., 2016; Gosset et al., 2020; Pu et al., 2020). The chemicals used are a flame retardant (perfluorooctanesulfonic acid [PFOS]), a commonly used anti‐inflammatory drug (diclofenac), the antibiotic trimethoprim, the herbicide atrazine and a heavy metal (arsenic). We use concentrations that are environmentally relevant based on literature research, opting for mid‐ to high environmental concentrations found either in high‐income or developing countries (Graziano et al., 2006; Le Luu, 2019).

*Daphnia* plays a central role in freshwater foodwebs worldwide (Altshuler et al., 2011). It has a parthenogenetic life cycle, in which sexual and asexual reproduction alternate (Ebert, 2005). Sexual recombination results in early‐stage embryos that arrest their development and enter dormancy, which can be exceptionally long (up to centuries; Kerfoot & Weider, 2004). Dormant embryos that are awakened (resurrected) from dormant stages are genetically distinct and can be propagated in the laboratory via clonal reproduction, allowing the rearing of populations of isogenic individuals (clones) from a single genotype (Cuenca‐Cambronero & Orsini, 2018). *Daphnia magna* strains were resurrected from the sedimentary archive of Lake Ring (Denmark), having a well‐documented history of exposure to chemicals and other stressors (Cuenca‐Cambronero et al., 2018; Davidson et al., 2007). By exposing strains that have never been exposed to chemical stress (naïve) and strains that have been historically exposed to chemical stress (experienced) to the five chemicals mentioned above, we were able to study how historical exposure to safe doses of chemicals in the natural environment can influence susceptibility to novel chemical stress. We tested the hypothesis that “experienced genotypes” were less susceptible to novel stress by showing higher fitness than “naive genotypes” when exposed to novel chemical stress. We expected experienced genotypes to have higher detoxification abilities underpinned by enriched detoxification genes or pathways, suggesting evolution of tolerance to chemical stress.

To test these hypotheses, we quantified fitness responses of two naïve and two experienced genotypes across three clonal generations to the five chemicals in common garden experiments. To link the observed changes in fitness to adaptive responses to chemicals, we quantified genome‐wide nucleotide diversity in the four genotypes and identified enriched functional pathways diverging between experienced and naïve genotypes. To investigate the relevance of our findings to other species, we used a comparative functional analysis of protein domains to identify enriched pathways in *Daphnia* that were conserved in other species and that are potential chemical targets. This study thus enabled us to investigate how evolutionary responses to novel chemical stress is potentially influenced by historical exposure to chemicals. We also gained insights into how the interactions between historical and novel chemical stress may influence transgenerational adaptive responses.

## 2 MATERIALS AND METHODS

### 2.1 Study system and experimental design

**FIGURE 1:**
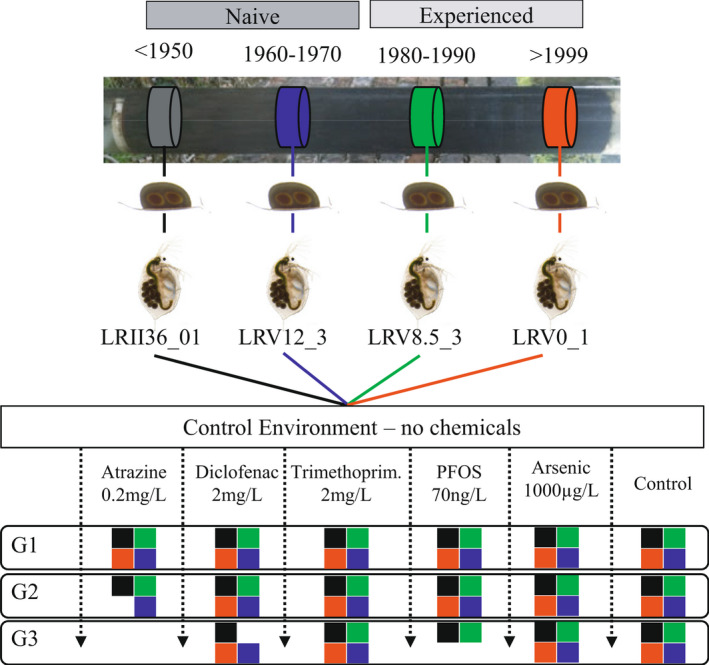
Experimental design. Four genotypes of *Daphnia magna*, previously resurrected from dormant embryos, were used for transgenerational exposures to five chemicals: PFOS (70 ng L^−1^), diclofenac (2 mg L^−1^), trimethoprim (2 mg L^−1^), atrazine (0.2 mg L^−1^) and arsenic (1,000 µg L^−1^). The four genotypes were resurrected from different times in the past, and were either “naïve” (black ‐ LRII36_1 [<1950] and blue, LRV12_3 [1960–1970]) or experienced (green, LRV8.5_3 [1980–1990] and red, LRV0_1 [>1999)] to chemicals. The clonal lines from the four genotypes were maintained in common garden conditions for at least two generations before the experiment to control for maternal effect. Five clonal replicates of each genotype, randomly selected from the control environment, were exposed to the five chemicals for three generations (G). Coloured squares represent genotypes at each generation (G). Some genotypes went extinct in G2 and G3

Genotypes of *Daphnia magna* were previously resurrected (revived) from a biological archive of Lake Ring, a shallow mixed lake in Denmark (55°57′51.83″N, 9°35′46.87″E) with a well‐documented history of anthropogenic impact (Cuenca‐Cambronero, Marshall, et al., 2018; Davidson et al., 2007). In the early 1900s and until late 1940s the lake was semipristine. In the late 1950s, it experienced eutrophication due to sewage inflow from a nearby town, which was diverted in the late 1970s; from the 1980s until the late 1990s, the lake experienced an increase in biocide run‐off due to agricultural land use intensification; the lake partially recovered from high nutrient levels in modern times but still received agricultural run‐off from the 1999s onward. According to these records, the lake experienced no chemical exposure until the 1970s, and high chemical exposure from the 1975s onward (Figure 1). Although the resident population of *D*. *magna* was probably exposed to multiple stressors over time, here we focus on tolerance to novel chemical stress and interpret fitness and functional responses to novel chemical stress in the context of historical chemical exposure. In our previous studies of the *D*. *magna* population from Lake Ring, we showed microevolutionary responses to recurrent chemical pollutants and that variance at fitness‐linked life history traits was larger between than within temporal populations (Cuenca‐Cambronero, Marshall, et al., 2018; Cuenca‐Cambronero, Marshall, et al., 2018; Cuenca‐Cambronero et al., 2018, 2021).

For the current study, a total of 360 exposures were completed. We selected a genotype from each of the four lake phases and exposed five clonal replicates per genotype across three clonal generations to six experimental conditions (five chemicals plus control; Figure 1). The genotypes are: LRII36_1 (<1950), LRV12_3 (1960–1970), LRV8.5_3 (1980–1990) and LRV0_1 (>1999). The former two genotypes are “naïve” and the latter two genotypes are “experienced” to chemical stress (Figure 1). The concentrations of the five chemicals used are as follows, reflecting environmentally relevant concentrations in surface waters: PFOS (70 ng L^−1^), atrazine (0.2 mg L^−1^), trimethoprim (2 mg L^−1^), diclofenac (2 mg L^−1^) and arsenic (1,000 µg L^−1^). The concentrations of PFOS and its derivatives vary dramatically between geographical areas and proximity to contamination sources (<0.8 ng L^−1^ to >17 mg L^−1^; Sinclair et al., 2006; Huang et al., 2010) but it is typically found at concentrations of 50–100 ng L^−1^ in surface waters (Bai & Son, 2021). Nonsteroidal anti‐inflammatory drugs such as diclofenac, acetaminophen and ibuprofen can be found at µg L^−1^ to g L^−1^ concentrations in seawater (Weigel et al., 2001) and surface waters (Fick et al., 2009), whereas their concentration is significantly lower in ground and drinking waters (ng L^−1^; Godfrey et al., 2007). A concentration between 1 and 2 mg L^−1^ is common in surface waters in high‐income economies (Fick et al., 2009). Average levels of antibiotic drugs in surface water range between ng L^−1^ and µg L^−1^ (Kummerer, 2009), with the exception of effluent originating from chemical manufacturers where concentrations of antibiotic drugs can exceed 30 mg L^−1^ (Larsson et al., 2007) and fish farms where they range from 1 to 6 mg L^−1^ (Le & Munekage, 2004). Atrazine is one of the most widely used photosynthesis‐inhibiting pre‐emergent biocides worldwide (Prado et al., 2014), with more than 70 million kg produced yearly (Sass & Colangelo, 2006). Typically, streams and rivers receiving agricultural run‐off display concentrations of 0.2 mg L^−1^ (Graziano et al., 2006). Metal contamination of groundwater is among the biggest health threats in low‐ and middle‐income countries (Winkel et al., 2011). According to the World Health Organization guidelines, the safe limit of arsenic in drinking water is 10 µg L^−1^ (EPA, 2001). In high‐income economies, arsenic in surface and urban waters typically reaches concentrations of 30 µg L^−1^ (Barrett et al., 2018). However, higher concentrations have been recorded occasionally in high‐income economies (e.g., North America; Smith et al., 2016) and frequently in low‐ and middle‐income countries (Chakraborti et al., 2018; Le Luu, 2019), where they range between 500 and 1,000 µg L^−1^ (Chakraborti et al., 2018). In some geographical regions, concentrations can exceed 3,000 µg L^−1^ (e.g., Vietnam; Le Luu, 2019).

### 2.2 Common garden experiments

We previously resurrected several genotypes of *D*. *magna* from Lake Ring and maintained them in the laboratory for over a year as isoclonal lines in the following standard laboratory conditions: 16:8‐hr light–dark photoperiod; 0.8 mg L^−1^ *Chlorella vulgaris* fed weekly; ambient temperature: 10°C (Cuenca‐Cambronero, Marshall, et al., 2018). Before the exposure experiment for this study, clonal lineages of the four genotypes (LRII36_1, LRV12_3, LRV8.5_3 and LRV0_1) were acclimatized for at least two generations to the following conditions to reduce interference from maternal effects: 16:8‐hr light–dark photoperiod; 0.8 mg L^−1^ *Chlorella vulgaris* fed daily; ambient temperature: 20°C. After at least two generations in these conditions, 24‐hr‐old juveniles from the second or following broods were randomly isolated and assigned to experimental conditions (Figure 1). Each clonal generation was established following the same criteria and starting from randomly selected 24‐hr‐old juveniles from the second or following broods. Where necessary, different broods from genotypes within the same generation were used to ensure developmental synchrony among clonal lineages in the experiment. Exposures of clonal lines were conducted in individual jars of 100 ml, filled with filtered borehole water (growth medium), which was refreshed every second day. The experimental cultures were fed daily *ad libitum* with 0.8 mg carbon L^–1^ of *Chlorella vulgaris*. To ensure constant concentrations of the chemicals throughout the experimental exposures, the growth medium was spiked with fresh chemicals at each media change. Stock solutions were prepared for all chemicals using pure ethanol as the carrier solvent. From this stock solution, dilutions of chemicals were prepared to the final concentrations listed above and a final concentration of ethanol not exceeding 0.005 ml L^–1^. The ethanol final concentration was previously shown to have a negligible effect on *Daphnia*, with patterns in life history traits not significantly departing from control conditions (Cuenca‐Cambronero, Marshall, et al., 2018; Jansen et al., 2015).

The following fitness‐linked life history traits were measured across three generations of four genotypes and five clonal replicates for exposed and nonexposed *Daphnia* (120 individuals across three clonal generations): age at maturity (first time parthenogenetic eggs are released in the brood pouch), size at maturity (distance from the head to the base of the tail spine), fecundity (sum of juveniles across two broods), interval between broods and mortality. Size at maturity was measured after the release of the first brood in the brood pouch using imagej software (https://imagej.nih.gov/ij/). The mortality rate per genotype was determined with the survival model fit using the psm function in the “rms” package in R version 3.6.0 (Harrell Jr, 2001). The day of mortality and mortality events were combined as a response variable while the term “genotype” was treated as a fixed effect. The mortality curves per generation were plotted with the survplot function from the rms package in R version 3.6.0 (Harrell Jr, 2001). Genotypes were fixed across all experimental conditions and clonal generations, enabling us to control for confounding factors, such as genetic changes occurring from one generation to the next and genetic variation among experimental exposures. Clonal replicates were nested within genotype in the statistical analyses as explained below. This design permits the analysis of within (WGP) and transgenerational plasticity (TGP), as well as the analysis of evolutionary differences among genotypes that originate from the same genetic pool.

### 2.3 Fitness response to chemicals

We quantified genetic, WGP and TGP using an analysis of variance (ANOVA), and tested the effect of Generation (G), Genotype (Gen), Treatment (T) and their interaction terms on the five fitness‐linked life history traits described above; a Wald chi‐square test (Type III test) was used to generate an analysis of deviance table (Langsrud, 2003). Multivariate effects were calculated on the same terms using multivariate statistics (MANOVA). Both statistics were completed using the “car” package for R version 3.6.0 (Fox & Weisberg, 2019) after checking for normality assumptions by plotting model residual vs. fitted values (Q‐Q plots) (Zuur et al., 2010). Clonal replicates were fit as a random effect nested within genotype. As the four genotypes used here belong to populations separated in time that originate from the same genetic pool, a significant genotype term indicates genetic evolution of the life history trait (Orsini et al., 2016). Differences in mean trait values between an individual treatment and its control within a generation are the expression of WGP. Differences in mean trait values across clonal generations are the expression of TGP. If the effect of the treatment differed significantly among genotypes (genetic effect), we would have evidence of a Gen (genotype) × T (treatment) interaction. Similarly, if the effect of the treatment differed significantly among generations, we would have evidence of a G (generation) × T (treatment) interaction. If genotype means varied by generation, we would have evidence of a G (generation) × Gen (genotype) interaction. We also measured the three‐way interaction term, which measures how the treatment per genotype differed across generations (Gen × G × T).

The main effects of genotype, treatment and generation plus their interaction terms on individual life history traits were visualized through univariate reaction norms, which describe the pattern of phenotypic expression of each genotype across treatments and generations (Roff, 1997). We visualized the multivariate analysis results using phenotypic trajectory analysis (PTA) plots to describe the difference in multivariate reaction norms in terms of magnitude and direction of change (Adams & Collyer, 2009). In the PTA, reaction norms are described as multivariate vectors with varying magnitude (the amount of phenotypic change between environments) and direction (the covariation of phenotypic variables) projected onto principal components in a multivariate space (Collyer & Adams, 2007). The R code provided by Adams and Collyer (2009) was used for the PTA. We visualized the principal mode of variation and covariation among traits (trade‐offs) within and across generations through a principal components analysis (PCA) done with the “prcomp” function in R followed by visualization through the fviz_pca_biplot function in the factoextra package in R version 3.6.0 on log‐transformed data (Kassambara & Mundt, 2020).

### 2.4 Genomics and functional analysis

We used clonal replicates of the four genotypes used in the exposure experiments to generate whole genome resequencing. The genotypes were cultured in standard laboratory conditions (16:8‐hr light–dark regime, 20°C and 0.8 mg C L^–1^ of *Chlorella vulgaris* daily) to generate sufficient material for extraction of high‐molecular‐weight genomic DNA (gDNA; typically 1 µg per library). Two days before tissue collection, cultures were treated with a cocktail of antibiotics at a final concentration of 20 mg L^−1^ (tetracycline [T], streptomycin [S], ampicillin [A]) to reduce bacterial contamination from gut microbes in downstream analyses. *Daphnia* were also deprived of food for 24 hr before tissue collection to reduce contamination from the feedstock (algae) in downstream analyses. gDNA was extracted using an Agencourt DNA Advance (Beckman Coulter; A48706). gDNA was quantified using a ND‐8000 Nanodrop (Thermo Fisher Scientific; ND‐8000‐GL). Up to 1 μg of gDNA per genotype was sheared using a Bioruptor Pico ultrasonicator with integrated cooling module (Diagenode; B01060010), following cooling on ice for 10 min. Sheared gDNA was assayed on a 2200 TapeStation (Agilent) with high‐sensitivity DNA screen tapes. The sheared gDNA was then prepared into Illumina‐compatible DNA 250‐bp paired‐end libraries using a KAPA HyperPrep Kit (Roche; KK8504), without amplification. Following library construction, libraries were assayed and quantified on a 2200 TapeStation (Agilent) with high‐sensitivity DNA screen tapes. Libraries were normalized to an average concentration of 2 nm prior to pooling. Libraries were sequenced on a HiSeq2500 (Illumina) using HiSeq Rapid SBS Kit version 2 200 cycles (Illumina; FC‐402–4021), HiSeq PE Rapid Cluster Kit version 2 (Illumina; PE‐402–4002) and HiSeq Rapid Duo cBot Sample Loading Kit (Illumina; CT‐403–2001) following the manufacturer's instructions aiming to reach a final depth of coverage of at least 40× per genotype.

The genome sequences were subjected to quality checking by mapping raw reads onto the newly assembled reference genome of *D*. *magna* obtained using a hybrid assembly of long and short reads—the detailed description of this reference genome will be presented elsewhere (NCBI: SUB9530054). Mapping and quality filtered single nucleotide polymorphism (SNP) variants were identified using the following steps: (i) read sequences base pair quality was assessed using fastqc (https://www.bioinformatics.babraham.ac.uk/projects/fastqc/) and multiqc (https://multiqc.info/); (ii) trimmomatic version 0.33 was applied for adapter trimming and to remove low‐quality sequences (Bolger et al., 2014). Paired‐end reads with Q > 30 and read length >50 bp were retained and mapped against the reference genome of *D*. *magna* using the BWA‐mem algorithm (Li & Durbin, 2010). (iii) samtools were used for format conversion, sorting, indexing and merging of mapping files onto the reference genome following (Li et al., 2009); (iv) picard tools were used to mark and remove polymerase chain reaction (PCR)‐duplicated reads and realign the data set with gatk (McKenna et al., 2010); (iv) allelic variants were called via bcftools (https://samtools.github.io/bcftools/) after applying the samtools mpileup command (samtools version 0.1.19, 45); and (vi) SNP were filtered with vcftools version 0.1.14. Filtering criteria were as follows: minimum read depth (DP) >10; SNP calls Quality (Phred score) for each sample Q > 30; minor allele frequency (MAF) >5%; maximum missing values 50%; Avg. genotype quality >50.

We studied genome‐wide and chromosomal‐level alpha and beta SNP diversity following methods described in Ma et al. (2020). We quantified gene‐level beta diversity between each pair of genotypes using noiseq with no replicates mode (Tarazona et al., 2015). We identified significant differences in SNP diversity per gene between each pair of genotypes using a probability approach (Tarazona et al., 2015). To understand the potential functional impact of the gene diversity differences among pairs of genotypes, we used a gene set enrichment analysis at multiple levels of functional categories including gene ontology and metabolic pathways. First, we used interproscan (Jones et al., 2014), which classifies genes into families, based on their protein sequence, and predicts their function based on domain information. interproscan uses predictive models provided by several databases including Pfam, PANTHER, CDD, GO, and KEGG. We used gprofiler (Raudvere et al., 2019) for gene ontology (GO) annotations and to perform a gene set enrichment analysis, using gene homology between *D*. *magna* and *D*. *pulex* (Colbourne et al., 2011). Based on the GO annotations, we identified enriched functional pathways using gprofiler (Raudvere et al., 2019). Both for enriched pathways and gene ontologies we used *p* <.05 after Bonferroni correction.

## 3 RESULTS

### 3.1 Fitness response to novel chemical stress of naïve and experienced genotypes

The five chemicals had a significant effect on the overall fitness of *Daphnia* genotypes. This effect was driven by different combinations of individual life history traits in the five chemical exposures. In the following, we describe the overall impact on fitness (MANOVA) and unpack the contribution of individual life history traits on this overall response by interpreting the ANOVA results.

A significant three‐way interaction term (Table 1; MANOVA, G × Gen × T) was observed, showing that treatment per genotype differed across generations in all chemicals, except for PFOS. ANOVA revealed that these overall patterns were driven by: (i) mortality in the PFOS exposure; (ii) size at maturity, age at maturity, fecundity and mortality in the atrazine exposure; (iii) size and age at maturity in the diclofenac exposure; (iv) size at maturity, fecundity and mortality in the trimethoprim exposure; and (v) fecundity age at maturity in the arsenic exposure (Table 1; ANOVA, G × Gen × T).

**TABLE 1:** Analysis of variance. Multivariate (MANOVA) and univariate (ANOVA) analysis of variance, testing the effect of generation (G), genotype (Gen), treatment (T) and their interaction terms on five fitness‐linked life history traits. The chemical tested are: PFOS (70 ng L^−1^), diclofenac (2 mg L^−1^), trimethoprim (2 mg L^−1^), atrazine (0.2 mg L^−1^) and arsenic (1,000 µg L^−1^). Size at maturity (mm), age at maturity (days), fecundity (number of offspring across two broods), interval between broods (time elapsed between broods) and mortality were measured. Each genotype is run in five clonal replicates. Significant *p*‐values are in bold

MANOVA				ANOVA											
					Size at maturity (mm)			Age at maturity (days)		Fecundity		Interval between broods		Mortality	
	df	*F*	*p*‐Value		df	Chi‐sq	*p*‐Value	Chi‐sq	*p*‐Value	Chi‐sq	*p*‐Value	Chi‐sq	*p*‐Value	Chi‐sq	*p*‐Value
*PFOS*															
Generation (G)	2	9.04	3.2e‐09***	Generation (G)	2	41.68	8.9e‐10***	41.94	7.8e‐10***	62.40	2.8e‐14***	8.10	.02*	6.48	.010891*
Genotype (Gen)	3	4.31	7.4e‐06***	Genotype (Gen)	3	5.14	.16	2.61	.46	29.19	2.0e‐06***	0.34	.95	16.16	.001052**
Treatment (T)	1	2.69	.04*	Treatment (T)	1	19.74	8.8e‐06***	0.49	.48	4.09	.04304*	0.03	.87	5.18	.022829*
G × Gen	4	1.59	.07	G × Gen	4	18.74	.0009***	9.17	.06	33.20	1.1e‐06***	1.29	.86	5.69	.13
G × T	2	3.07	.004**	G × T	2	13.29	.001**	23.12	9.5e‐06***	7.44	.02*	1.72	.42	1.87	.17
Gen × T	3	1.94	.03*	Gen × T	3	22.85	4.3e‐05***	0.54	.91	1.28	.73	1.92	.59	8.98	.029603*
G × Gen × T	4	1.19	.27	G × Gen × T	4	7.03	.13	1.52	.82	4.45	.35	1.29	.86	8	.038836*
*DICLOFENAC*															
Generation (G)	2	14.77	2.1e‐15***	Generation (G)	2	73.63	<2.2e‐16***	110.90	<2.2e‐16***	127.47	<2.2e‐16***	21.35	2.3e‐05***	7.85	.0197461*
Genotype (Gen)	3	2.67	.002**	Genotype (Gen)	3	13.25	.004**	6.34	.10	29.37	1.9e‐06***	0.50	.92	7.76	.05
Treatment (T)	1	16.24	2.4e‐09***	Treatment (T)	1	44.52	2.5e‐11***	39.40	3.4e‐10***	16.32	5.3e‐05***	0.12	.73	13.45	.0002447***
G × Gen	5	2.37	.001**	G × Gen	5	26.05	8.7e‐05***	17.25	.004**	56.96	5.2e‐11***	3.43	.63	14.84	.0110460*
G × T	2	10.81	1.0e‐11***	G × T	2	31.09	1.8e‐07***	83.62	<2.2e‐16***	21.13	2.6e‐05***	5.76	.06	21.33	2.331e‐05***
Gen × T	3	2.59	.003**	Gen × T	3	13.52	.004**	5.42	.14	2.00	.57	2.82	.42	1.97	.58
G × Gen × T	5	3.51	1.370e‐06***	G × Gen × T	5	25.37	.0001***	17.25	3.5e‐07***	8.84	.12	5.35	.37	0	1
*TRIMETHOPRIM*															
Generation (G)	2	16.77	<2.2e‐16***	Generation (G)	2	16.65	.0002***	87.14	<2.2e‐16***	92.88	<2.2e‐16***	21.85	1.8e‐05***	0.10	.76
Genotype (Gen)	3	4.35	.55	Genotype (Gen)	3	18.29	.0004***	18.29	.0003827***	39.90	1.1e‐08***	1.35	.72	7.01	.14
Treatment (T)	1	1.45	.22	Treatment (T)	1	0.25	.62	0.34	.56	0.07	.79	4.20	.04*	0.94	.33
G × Gen	6	2.31	.0006***	G × Gen	6	21.26	.002**	17.84	.006637**	28.44	7.76e‐05***	4.19	.65	2.76	.43
G × T	2	2.30	.023*	G × T	2	0.90	.64	7.55	.022895*	4.17	.12	1.61	.45	0.04	.85
Gen × T	3	3.39	.0001***	Gen × T	3	29.47	1.8e‐06***	3.78	.29	16.34	.0001***	2.43	.49	2.63	.45
G × Gen × T	6	1.75	.02*	G × Gen × T	6	16.89	.01**	9.25	.16	12.92	.04*	7.95	.24	9.23	.02639*
*ATRAZINE*															
Generation (G)	2	7.07	.84	Generation (G)	2	44.74	1.9e‐10***	39.83	2.2e‐09***	35.42	2e‐08***	3.88	.14	0.01	.90
Genotype (Gen)	3	1.50	.39	Genotype (Gen)	3	23.06	3.9e‐05***	3.98	.26	21.28	9.2e‐05***	0.82	.84	12.87	.004920**
Treatment (T)	1	15.46	1.4e‐07***	Treatment (T)	1	89.50	<2.2e‐16***	0.52	.47	33.65	6.6e‐09***	0.18	.68	20.53	5.881e‐06***
G × Gen	2	2.49	.02*	G × Gen	2	10.06	.006**	2.35	.31	8.69	.01294*	0.12	.94	4.38	.11
G × T	2	2.19	.04*	G × T	2	6.42	.04*	3.55	.17	1.62	.44	0.67	.71	0.14	.71
Gen × T	3	2.24	.55	Gen × T	3	4.18	.24	3.84	.28	1.73	.63	1.54	.67	4.96	.18
G × Gen × T	1	2.76	.04*	G × Gen × T	2	7.12	.03*	2.84	.03*	6.52	.04*	0.00	.99	10.2274	.006014**
*ARSENIC*															
Generation (G)	2	14.17	4.233e‐15***	Generation (G)	2	83.76	<2.2e‐16***	39.14	3.165e‐09***	0.35	.84	8.26	.0161066*	2.53	.28
Genotype (Gen)	3	4.15	7.332e‐06***	Genotype (Gen)	3	17.58	.0005378***	134.18	<2.2e‐16***	38.44	2.284e‐08***	6.25	.10	1.01	.80
Treatment (T)	1	1.24	.30	Treatment (T)	1	1.81	.18	1.91	.17	0.03	.85	0.08	.77	0.00	.98
G × Gen	6	6.08	3.067e‐15***	G × Gen	6	66.16	2.501e‐12***	59.61	5.394e‐11***	22.31	.001066**	27.59	.0001124***	11.66	.07
G × T	2	3.03	.003557**	G × T	2	10.83	.0044538**	8.50	.01425*	2.75	.25	1.34	.51	7.81	.020177*
Gen × T	3	2.61	.002843**	Gen × T	3	9.15	.0273738*	10.75	.01314*	3.24	.36	1.34	.72	11.64	.008706**
G × Gen × T	6	3.23	1.387e‐06***	G × Gen × T	6	11.75	.07	38.13	1.061e‐06***	33.35	8.985e‐06***	1.33	.97	0	1

Exposure to the chemicals induced a significant genetic response both within (Gen × T) and between (G × Gen) generations. As the genotypes originate from the same genetic pool sampled at different times in the past, a significant difference in mean trait values among genotypes indicates evolutionary differences. A genotype‐dependent response to treatment was observed in all chemicals, except for atrazine (Table 1; MANOVA, Gen × T). The individual fitness‐linked life history traits contributing to this response differed among treatments: (i) size at maturity and mortality significantly varied between genotypes in the PFOS treatment; (ii) size at maturity varied by genotype in the diclofenac exposure; (iii) size at maturity and fecundity varied among genotypes in the trimethoprim exposure; and (iv) size and at maturity, and mortality varied among genotypes in the arsenic treatment (Table 1; ANOVA, Gen × T).

A significant genotype per generation effect (G × Gen) was observed across treatments, except for PFOS (Table 1; MANOVA, G × Gen). The individual fitness‐linked life history traits contributing to this overall fitness response were: (i) size at maturity and fecundity for PFOS; (ii) size at maturity and fecundity for atrazine; (iii) all traits except interval between broods for diclofenac; (iv) size at maturity, age at maturity and fecundity for trimethoprim; and (v) all life history traits except mortality for arsenic (Table 1; ANOVA, G × Gen).

Plasticity was pervasive within and across generations. Significant plastic responses to treatment (T) within generations, indicative of WGP, were observed in PFOS, atrazine and diclofenac (Table 1; MANOVA, T). These plastic responses induced: (i) a significant decline in size at maturity and an increase in mortality in PFOS exposures; (ii) a significant decline in size at maturity, and an increase in fecundity and mortality in the atrazine exposures; (iii) a decline in size at maturity, a delay in maturation, a decline in fecundity and higher mortality in the diclofenac exposures; and (iv) a genotype‐specific change in the time between broods in the trimethoprim exposure (Table 1; ANOVA, T; Figures S1 and S2). The WGP effects on individual life history traits are summarized through univariate reaction norms in Figure S1. An overview of mortality across treatments and generations is presented in Figure S2.

Significant TGP was observed in all exposures (Table 1, G × T). These plastic responses had a significant effect on: (i) size at maturity, age at maturity and fecundity in PFOS exposures; (ii) size at maturity in atrazine exposure; (iii) size at maturity, age at maturity, fecundity and mortality in diclofenac exposures; (iv) age at maturity in trimethoprim exposures; and (v) age at maturity, size at maturity and mortality in arsenic exposures (Table 1; ANOVA, G × T).

**FIGURE 2:**
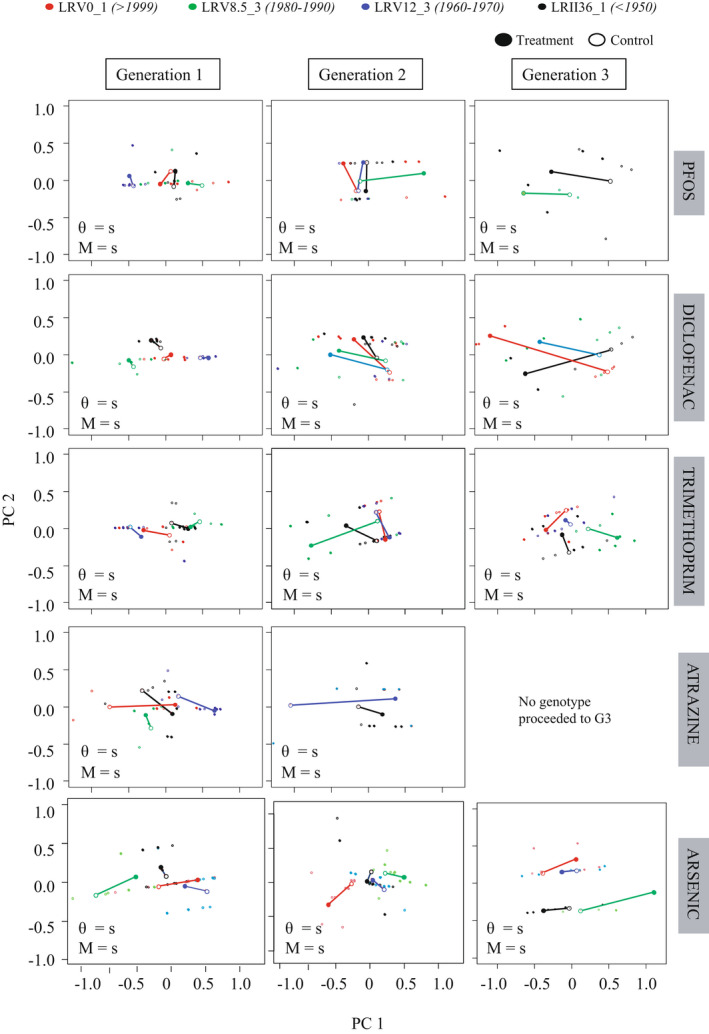
Phenotypic trajectory analysis. PTA on the four genotypes of *Daphnia magna* used in transgenerational exposure to five chemical classes (PFOS [70 ng L^−1^], diclofenac [2 mg L^−1^], trimethoprim [2 mg L^−1^], atrazine [0.2 mg L^−1^] and arsenic [1,000 µg L^−1^]), resulting from multivariate response of five fitness‐linked life history traits. Open circles represent the control (nonexposed clonal replicates) and full circles represent the exposed clonal replicates. Genotype centroids are connected by reaction norms (solid lines), showing phenotypic change in direction and length. Differences among genotypes in terms of magnitude (M) and direction (θ) of plastic response are all significant. The statistics supporting the PTA are given in Table S1. Genotypes are colour‐coded as in Figure 1

TGP effects visualized through PTA plots revealed an overall trend of lower mortality and smaller fitness change across clonal generations in naïve than in experienced genotypes. It also revealed different overall fitness responses of the four genotypes. Exposure to PFOS caused an increasing magnitude of change in all four genotypes as the generations progressed and change in direction for the naïve genotype LRII36_1 in the third generation (Figure 2, PFOS; Table S1). In the first generation of this exposure, the experienced genotype LRV0_1 showed a negative fitness change. In the second generation, the naïve genotype LRII36_1 showed a negative fitness change. Two of the four genotypes (LRV0_1 and LRV12_3) went extinct in generation three (Figure 2; PFOS). Exposure to diclofenac induced changes in both the magnitude and direction of fitness across the genotypes (Figure 2, diclofenac). However, whereas in generation 2 all genotypes experienced an increase in fitness (the treatment had higher fitness than the control), this pattern was reversed for the naïve genotype LRII36_1 in generation 3, where we also observed mortality of the experienced genotype LRV8.5_3 (Figure 2, diclofenac). Exposure to trimethoprim induced genotype‐specific fitness changes in both direction and magnitude (Figure 2, trimethoprim). In the third generation, the naïve genotypes LRV12_3 and LRII36_1 experienced a positive change in fitness (Figure 2, trimethoprim). Exposure to atrazine imposed the highest fitness costs across the genotypes, resulting in the mortality of the experienced genotypes in generation 2 and of all genotypes in generation 3 (Figure 2, atrazine). This exposure induced changes in both the direction and magnitude of fitness in the first generation, with the naïve genotype LRII36_1 showing the least change in fitness between the two naïve genotypes that survived the first generation (Figure 2, atrazine). Exposure to arsenic induced genotype‐specific changes across generations; the naïve genotypes (LRV12_3 and LRII36_1) experienced the smallest overall fitness change (Figure 2, arsenic).

### 3.2 Detoxification pathways and genome‐wide diversity in naïve and experienced genotypes

**FIGURE 3:**
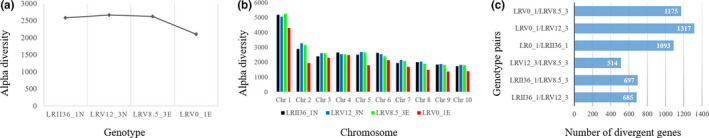
Genomic diversity. Alpha diversity measured at (a) genome‐wide and (b) chromosome‐level in the four genotypes used in this study; (c) number of significantly divergent genes between each pair of genotypes. The genotypes are colour coded as in Figure 1: LRII36_1 (<1950; black), LRV12_3 (1960–1970; blue), LRV8.5_3 (1975–1985; green) and LRV0_1 (> 1999; red).

The genome of the four genotypes of *D*. *magna* was assembled (NCBI: SUB9530054) and the raw depth of coverage was: 84× for LRV0_1; 42× for LRV8.5_3; 49× for LRV12_3; and 45× for LRII36_1. The genome‐wide SNP‐alpha diversity was comparable among the genotypes LRII36_1, LRV12_3 and LRV8.5_3, and lower in the most recent genotype (LRV0_1) (Figure 3a; Table S2). The genome‐wide alpha diversity patterns were reflected equally across chromosomes (Figure 3b). Beta diversity was significant in all pairwise comparisons but comparatively higher in pairwise comparisons including the LRV0_1 genotype (Table S2; *p* =.05).

The number of divergent genes between LRV0_1 and the other genotypes ranged between 1,093 (3.3% of the total number of *Daphnia* genes) and 1,317 (4%). Conversely, the number of significantly divergent genes in the pairwise comparisons involving the other genotypes ranged between 514 (1.6% of the total number of *Daphnia* genes) and 697 (2.1%) (Figure 3c; Table S3). The mean gene diversity at the divergent genes was significantly lower in the experienced than in the naïve genotypes (*t* test; *p* =.03).

To understand the potential functional impact of the divergent genes between naïve and experienced genotypes, we used a gene set enrichment analysis at different functional levels including gene ontology and metabolic pathways. Divergent genes were enriched for detoxification (Cytochrome P450), catabolite degradation (Armadillo), exoskeletal modelling (e.g., chitin‐binding domain), fertilization (Alpha‐l‐fucosidase) and embryonic development (Nuclear hormone receptor‐like domain superfamily, Zona pellucida domain) (Table S4). Genes involved in endocrine processes (GPCR), epigenetic regulation (the Histone deacetylase family and the Elongator complex protein 1) and neuronal activities (learning and memory) were also enriched (Table S4). Gene enrichment analysis also identified significant change in genes underpinning fundamental cell functions and trafficking: oxidoreductase activity, redox and biosynthetic reactions (Dehydrogenase/reductase, Haem peroxidase), heat shock proteins (HSP70), DNA binding (Rap1 Myb domain, Zinc finger, MIZ‐type), recombination (RAD50, zinc hook), and replication/cell division (Creatinase/aminopeptidase‐like, Mad3/Bub1, DNA mismatch repair protein MutS, WD40), protein–protein interactions (Ankyrin) and cell trafficking (Dynamin, von Willebrand factor) (Table S4).

We used the manually curated “Reactome” database to assess how many of the functional pathways diverging between naïve and experienced genotypes in our study were conserved across other animals, based on conservation of the protein sequence (Table S4). These domains could be potential targets of chemical pollution in other species: (i) genotoxic stress response and detoxification functions (R‐HSA‐176187, R‐HSA‐3299685, R‐HSA‐8849175); (ii) catabolism (R‐HSA‐71064, R‐HSA‐389661, R‐HSA‐70370); (iii) immunity genes including the major histocompatibility complex (R‐HSA‐6798695, R‐HSA‐2132295, R‐HSA‐2132295); (iv) fat and carbohydrate metabolism (R‐HSA‐8964038, R‐HSA‐1369062, R‐HSA‐2024096, R‐HSA‐70171, R‐HSA‐189085, R‐HSA‐189200, R‐HSA‐1655829, R‐HSA‐351202, R‐HSA‐1369062); and (v) nervous system (R‐HSA‐8862803, R‐HSA‐375165), including the neurotransmitter acetylcholine (R‐HSA‐6798163). Conserved domains across species also included DNA repair (R‐HSA‐1221632, R‐HSA‐2559586, R‐HSA‐5632928) and elongation (R‐HSA‐73980); RNA binding, translation and transcription, including miRNA (R‐HSA‐72702, R‐HSA‐156827, R‐HSA‐203927, R‐HSA‐4641265); histone demethylation (R‐HSA‐3214842); and cell signalling, including transcription factors that bind hormones and vitamins (R‐HSA‐913709, R‐HSA‐391160, R‐HSA‐5663220, R‐HSA‐383280, R‐HSA‐2682334, R‐HSA‐2565942, R‐HSA‐425561, R‐HSA‐2682334) (Table S4). Fat and carbohydrate metabolism pathways were differentially enriched between “naive” and “experienced” genotypes (Table S5).

## 4 DISCUSSION

### 4.1 Naïve genotypes show higher fitness in response to novel chemical stress

We hypothesized that experienced genotypes had an evolutionary advantage over naïve genotypes when exposed to novel chemical stress. We expected the experienced genotypes to always have higher overall fitness underpinned by enrichment at detoxification genes or pathways.

Our results show significant fitness differences among genotypes, underpinned by divergence in functionally enriched pathways for detoxification, catabolic and metabolic functions between experienced and naïve genotypes. However, naïve genotypes showed a higher tolerance to novel chemical stress, and lower fitness costs measured via ecological endpoints (e.g., mortality) and phenotypic trajectory changes, confuting our hypothesis. Previous studies on parthenogenetic species that experienced different levels of anthropogenic stress reached similar conclusions (e.g., rotifers: Zweerus et al., 2017; other *Daphnia* species: Spaak & Keller, 2004).

ANOVA on the fitness‐linked life history traits revealed that the evolutionary mechanisms underpinning fitness responses to novel chemical stress were complex. All four genotypes expressed both WGP and TGP to cope with novel chemical stress. However, whereas WGP enabled both naïve and experienced genotypes to respond to novel chemical stress in the short term, TGP affected naïve and experienced genotypes differently. This short‐term strategy was evident from the mortality of the experienced genotypes in 60% of the chemicals tested between the second and the third generation. Where mortality did not occur (e.g., trimethoprim and arsenic), the experienced genotypes showed larger fitness changes than the naïve genotypes as the generations progressed. These fitness changes occurred between the second and the third generation of exposure (PFOS, atrazine and diclofenac), indicating cumulative toxicity effects. A complex interplay between genetic adaptation, WGP and TGP has been previously observed in population‐level studies of the *Daphnia* population from Lake Ring, suggesting that the genotypes used in this study are representative of the *Daphnia* subpopulations from Lake Ring (Cuenca‐Cambronero, Marshall, et al., 2018; Cuenca‐Cambronero et al., 2021; Toyota et al., 2019). A population‐level analysis will be required to confirm the observed patterns in our study.

Highly plastic traits tend to show strong maternal effect variance and little to no genetic variance, because they are more strongly influenced by the environment, including parental environment, and because additive genetic variance may be masked by high environmental variation (Donelan et al., 2020). In our study, the average fitness of the genotypes increased in the second generation, but it declined again in the third generation, indicating transient positive maternal effects. Transient positive maternal effects have been observed in transgenerational studies of *D*. *magna* exposed to gamma radiation (Parisot et al., 2015). Conversely, a persistent positive maternal effect has been observed in transgenerational studies on photoperiod length (Toyota et al., 2019) and endocrine disruptors (Clubbs & Brooks, 2007; Tanaka & Nakanishi, 2002). A positive maternal effect is experienced when the offspring environment perfectly matches the maternal one. In human‐altered environments, such as the one linked to chemical run‐off, it may be more difficult for the parental generation to detect and correctly identify novel environmental conditions that lack historical context or that increase environmental variability. In these conditions the parents may fail to respond because they lack appropriate cue–response systems (Burgess et al., 31. 2014). The transient maternal effects observed in our study could be explained by epigenetic mechanisms, as suggested by the lower diversity at epigenetic regulation genes (Histone deacetylase family and the Elongator complex protein 1) in experienced genotypes. Previous epigenetic studies on cross‐generation exposures of *Daphnia* to gamma radiation identified small but significant differential methylation that could explain transgenerational transient effects in fitness (Parisot et al., 2015; Trijau et al., 2018). However, given the low genome‐wide methylation in *Daphnia*, it was not possible to unequivocally link fitness and epigenetic changes (Trijau et al., 2018). Cross‐generational transient fitness effects can be also explained by compensatory mechanisms, expressed through trade‐offs among life history traits (Cuenca‐Cambronero et al., 2021).

### 4.2 Higher fitness in naïve genotypes is underpinned by higher diversity in detoxification genes

Our findings reject the hypothesis that “experienced genotypes” have an evolutionary advantage in the presence of novel chemical stress. In our seminal study on the *Daphnia* population from Lake Ring, the experienced genotypes showed a comparatively higher fitness when exposed to the same chemicals recorded in the historical environment, even if this was dependent on the severity of the stress (Cuenca‐Cambronero, Marshall, et al., 2018). The limitation of our study is in the small number of clones used, which may not be representative of the local population genetic diversity, therefore providing a qualitative rather than quantitative support to our hypothesis testing. However, if the patterns observed here are validated at the population level, the results of our study and of past studies on the Lake Ring *Daphnia* population suggest that the acquired tolerance to chemical stress may be evolutionarily advantageous to recurring but not novel chemical stress. It is noteworthy that one of the two naïve genotypes showed the smallest trade‐offs and an overall highest fitness (LRII 36_1) when exposed to novel chemical stress. This may be explained by this genotype being resurrected from a semipristine environment whereas the naïve genotype LRV12.3 was historically exposed to other stressors (e.g., eutrophication). It has been shown that multiple stressors linked to anthropogenic activities can influence how organisms adapt and evolve, with evolutionary mechanisms underpinning multiple stress responses being often synergistic (Cuenca‐Cambronero et al., 2021; Jackson et al., 2016; Orr et al., 2021).

Lower tolerance to novel chemical stress was associated with reduced genome‐wide genetic diversity in the modern genotype (LRV_1). These patterns suggest that genetic erosion occurred as result of multidecadal exposure to chemical stress (Diez‐Del‐Molino et al., 2018). The theory that genetic erosion occurred in Lake Ring has to be validated through population‐level analysis of genome‐wide variation. However, the significant decline in genetic diversity in the most modern genotype (LRV0_1), which was exposed to chemical pollution for 10–15 years/sexual generations (starting from the 1980s and accounting for a sexual generation per year in the *Daphnia* population), agrees with experimental studies on genetic erosion, showing a decline in genetic diversity following >12 generations of exposure to chemicals (e.g., Nowak et al., 2009). Our findings on pervasive plasticity both within and between generations align with the hypothesis of genetic erosion. Previous studies have shown that plasticity can be maintained in the face of genetic erosion, but it comes with fitness costs (Luquet et al., 2011) and with reduced tolerance to environmental stress (Bijlsma & Loeschcke, 2012).

Genes significantly divergent between naïve and experienced genotypes showed lower diversity in experienced genotypes. These genes were enriched for detoxification, catabolism and endocrine processes, as well as for embryonic development and epigenetic regulation. Fat and sugar metabolism pathways were divergent between naïve and experienced genotypes. These functional differences probably explain the lower overall fitness and higher mortality of the experienced genotypes in the exposures experiment. Some of the gene domains significantly enriched in *Daphnia* were conserved across the Tree of Life and included genotoxic stress response and detoxification functions, immunity genes and genes involved in nervous system functionality including the neurotransmitter acetylcholine. Although conservation of function does not imply identical biological outcomes, our results suggest that these pathways may be targets of toxicity in other species. These discoveries can inform (eco)toxicology assessments of persistent chemicals.

Overall, our study shows that exposure to chemical stress in *Daphnia* may impose costs associated with susceptibility to novel chemical stress, potentially compromising the resilience and adaptation to future environmental changes. Susceptibility may be influenced by the combined effect of other stressors interacting with chemicals, with potentially more severe effects. The impact on the keystone grazer *Daphnia* has important implications for aquatic food webs, given its central role in lentic freshwater environments worldwide. Functional pathways conserved across species and putatively disrupted by prolonged chemical exposure included detoxification, neuronal functions and metabolism. These pathways are potential targets in other species, including humans.

## CONFLICT OF INTERESTS

The authors declare no competing interests.

## AUTHOR CONTRIBUTIONS

M.A. performed the experiments. M.A. and J.Z. performed statistical analyses on fitness‐linked life history traits. V.D. and A.C. performed genomics and functional analysis. L.O. conceived the study, coordinated data analysis and writing. All authors contributed to the manuscript writing.

### OPEN RESEARCH BADGES

This article has earned an Open Data Badge for making publicly available the digitally‐shareable data necessary to reproduce the reported results. The data is available at https://doi.org/10.5061/dryad.4xgxd2591.

## Supporting information

Supplementary Material

Table S3

Table S4

## Data Availability

The genome sequence data can be found at BioProject ID PRJNA727483 in the NCBI repository. The fitness‐linked life history traits can be found in the dryad database at https://doi.org/10.5061/dryad.4xgxd2591.
