# Supplementary material for: Historical exposure to chemicals reduces tolerance to novel chemical stress in Daphnia (waterflea)
Source: Mol Ecol. 2022 Apr 15;31(11):3098–111. doi: 10.1111/mec.16451 (PMC9321109; doi:10.1111/mec.16451)

**Historical exposure to chemicals reduces tolerance to novel chemical stress in *Daphnia* (waterflea)**

Muhammad Abdullahi^1^, Jiarui Zhou^1^, Vignesh Dandhapani^1^, Anurag Chaturvedi^1^ and Luisa Orsini^1,2*^

^1^*Environmental Genomics Group, School of Biosciences, the University of Birmingham, Birmingham, B15 2TT, UK*

*^2^The Alan Turing Institute, British Library, 96 Euston Road, London NW1 2DB, UK.*

Contact information:

Luisa Orsini

l.orsini@bham.ac.uk

+44(0)1214145894

Running title: Resilience to novel chemical stress

**Table S1. PTA results.** Phenotypic trajectory analysis testing magnitude (magnitude) and direction (θ) of change across genotypes and generations for exposures to five chemicals: PFOS (70 ng/L); Diclofenac (2 mg/L); Trimethoprim (2 mg/L); Atrazine (0.2 mg/L) and Arsenic (1,000 µg/L). Missing data reflect genotypes’ extinction. Significant *P-values* are in bold. The statistics in this table support Figure 2.

|  | Generation 1 | | | | Generation 2 | | | | Generation 3 | | | |
| --- | --- | --- | --- | --- | --- | --- | --- | --- | --- | --- | --- | --- |
|  | Magnitude | *P*_mag_ | θ | *P*_θ_ | Magnitude | *P*_mag_ | θ | *P*_θ_ | Magnitude | *P*_mag_ | θ | *P*_θ_ |
| **PFOS** | 0.0007 | **0.002** | 1572.35 | **0.001** | 0.0600 | **0.001** | 1537.10 | **0.001** | 0.0000 | **0.002** | 0.00 | **0.001** |
| **DICLOFENAC** | 0.0068 | **0.001** | 1164.53 | **0.001** | 0.0125 | **0.002** | 274.51 | **0.001** | 0.0003 | **0.002** | 1418.90 | **0.001** |
| **TRIMETHOPRIM** | 0.0075 | **0.001** | 2481.88 | **0.001** | 0.0197 | **0.001** | 425.39 | **0.001** | 0.0197 | **0.001** | 425.39 | **0.001** |
| **ATRAZINE** | 0.0423 | **0.001** | 1720.02 | **0.001** | 0.0000 | **0.002** | 0.00 | **0.001** | n/a |  | n/a |  |
| **ARSENIC** | 0.0178 | **0.001** | 1675.63 | **0.001** | 0.0197 | **0.001** | 216.50 | **0.001** | 0.0882 | **0.001** | 6353.09 | **0.001** |
|  |  |  |  |  |  |  |  |  |  |  |  |  |

**Table S2. Alpha and beta diversity.** Chromosomal-level and genome-wide alpha diversity; and chromosomal-level and genome-wide beta diversity between each pair of genotypes used in the study (LRII36_1; LRV12_3; LRV8.5_3; and LRV0_1) supported by a Wilcoxon test.

|  |  | Alpha diversity | |  |  |  |  | Beta diversity | |  |  |
| --- | --- | --- | --- | --- | --- | --- | --- | --- | --- | --- | --- |
|  | LRV0_1 | LRV8.5_3 | LRV12_3 | LRII36_1 |  | LRV0_1 x LRV8.5_3 | LRV0_1 x LRV12_3 | LRV0_1 x LRII36_1 | LRV8.5_3 x LRV12_3 | LRV8.5_3x LRII36_1 | LRV12_3 x LRII36_1 |
| Chr1 | 4307 | 5266 | 5064 | 5192 | Chr1 | 1.13 | 1.12 | 1.13 | 1.08 | 1.06 | 1.09 |
| Chr2 | 1964 | 3176 | 3282 | 2891 | Chr2 | 1.26 | 1.27 | 1.23 | 1.05 | 1.09 | 1.10 |
| Chr3 | 2313 | 2605 | 2616 | 2408 | Chr3 | 1.10 | 1.11 | 1.11 | 1.05 | 1.11 | 1.11 |
| Chr4 | 2489 | 2524 | 2553 | 2662 | Chr4 | 1.10 | 1.14 | 1.10 | 1.11 | 1.11 | 1.11 |
| Chr5 | 1813 | 2653 | 2695 | 2519 | Chr5 | 1.24 | 1.23 | 1.21 | 1.07 | 1.12 | 1.11 |
| Chr6 | 2137 | 2406 | 2555 | 2630 | Chr6 | 1.13 | 1.13 | 1.14 | 1.10 | 1.10 | 1.09 |
| Chr7 | 1701 | 2079 | 2159 | 1966 | Chr7 | 1.14 | 1.15 | 1.14 | 1.05 | 1.10 | 1.09 |
| Chr8 | 1496 | 1911 | 2045 | 2009 | Chr8 | 1.19 | 1.18 | 1.18 | 1.07 | 1.07 | 1.05 |
| Chr9 | 1384 | 1837 | 1889 | 1847 | Chr9 | 1.18 | 1.19 | 1.18 | 1.07 | 1.07 | 1.08 |
| Chr10 | 1403 | 1819 | 1825 | 1734 | Chr10 | 1.15 | 1.16 | 1.15 | 1.06 | 1.07 | 1.08 |
| genome-wide | 2100.70 | 2627.60 | 2668.30 | 2585.80 | genome-wide | 1.16 | 1.17 | 1.16 | 1.07 | 1.09 | 1.09 |
| mean | 2101 | 2628 | 2668 | 2586 | mean | 1.16 | 1.17 | 1.16 | 1.07 | 1.09 | 1.09 |
| st. dev. | 1880 | 2364 | 2429 | 2325 | P-Val | 0.05 | 0.05 | 0.04 | 0.02 | 0.02 | 0.02 |

**Table S3. Divergent genes between pairs of genotypes.** Number of genes showing significant differences in SNP diversity between each pair of genotypes. The *D. magna* gene ID; the number of SNPs found in each gene in the pairwise comparison; the gene length in bp; the start and end position of the gene; the P-value supporting divergent number of SNPs per genes; and the Chromosome where the genes sit, are shown.

See Abdulahi *et al.*_TableS3.xls

**Table S4. Domain functional analysis.** List of *D. magna* genes (*D. magna*_gene ID) enriched in the pairwise genotype analysis identifying divergent genes; gene sequence length (bp); online database searcher for onthology (database); the functional analysis is based on curated databases, including Interproscan, Pfam, and Panther; start and end location of each gene; Interproscan ID; GO terms (GO ID); functional pathways conserved across the Tree of Life are listed from the Reactome database.

See Abdullahi *et al*_Table S4

**Table S5. Pathway analysis.** List of pathways enriched between genotypes from the chemical-free and chemical-loaded environment.

| Pathway function | Pthway ID | Adj P-val | Domain size |
| --- | --- | --- | --- |
| Lysosome | KEGG:04142 | 0.00 | 122 |
| Glycan degradation | KEGG:00511 | 0.01 | 122 |
| Lipids metabolism | KEGG:00130 | 0.02 | 46 |

**Figure S1. Cross-generational fitness burden of chemical pollution.** Univariate reaction norms of fitness-linked life history traits after exposure to five chemicals: [PFOS (70ng/L); diclofenac (2mg/L); trimethoprim (2 mg/L); atrazine (0.2 mg/L) and arsenic (1,000 µg/L)] across three clonal generations. Size at maturity (mm), age at maturity (days), fecundity, interval between broods (time elapsed between broods averaged over two broods) are shown. Average and SD per genotype across five biological replicates are shown. Genotypes are color-coded as in Figure 1: LRII36_1 (<1950; black); LRV12_3 (1960–1970; blue); LRV8.5_3 (1975–1985; green) and LRV0_1 (> 1999; red). Where reaction norms are not displayed, the genotypes went extinct in the treatment.


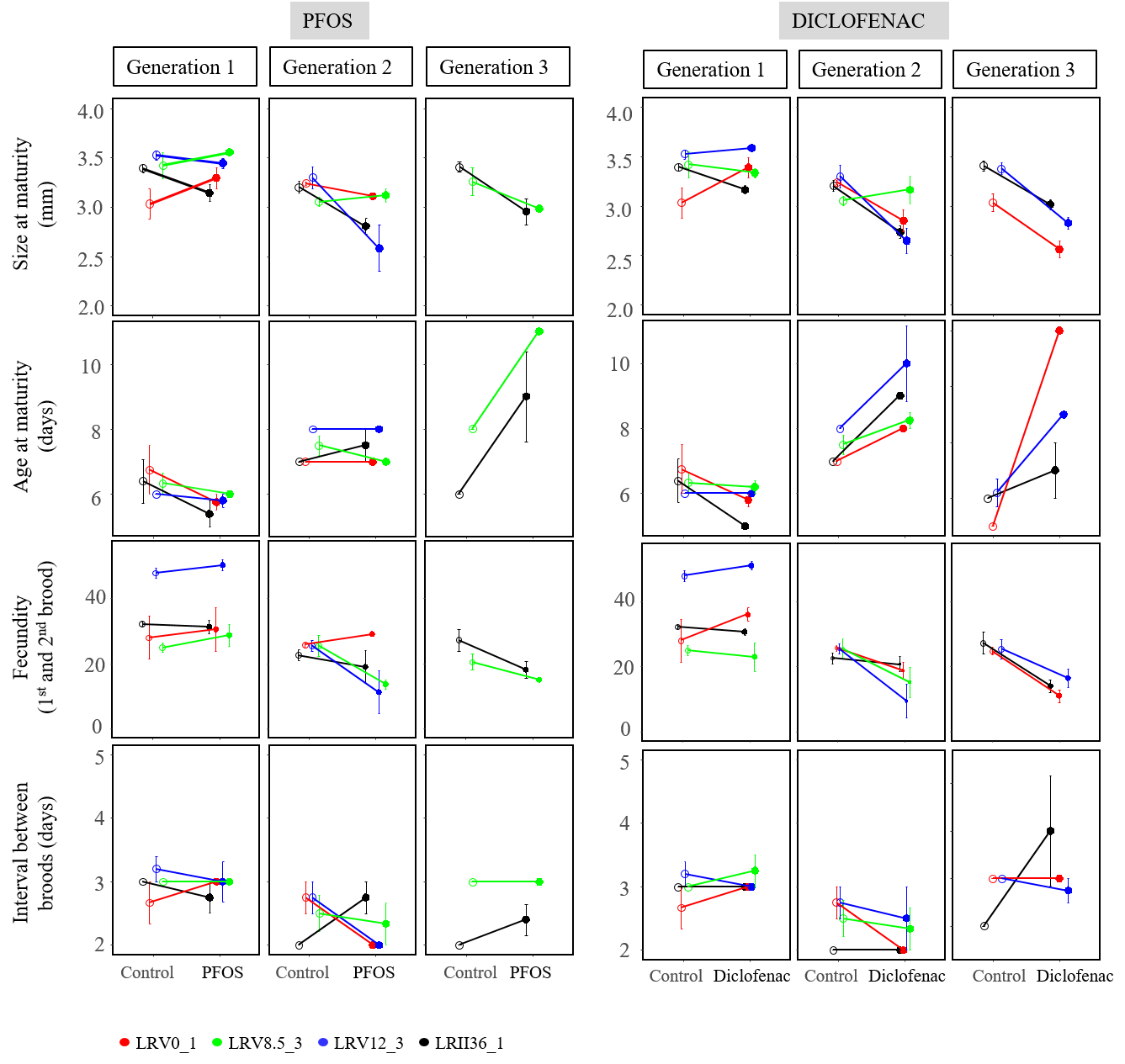

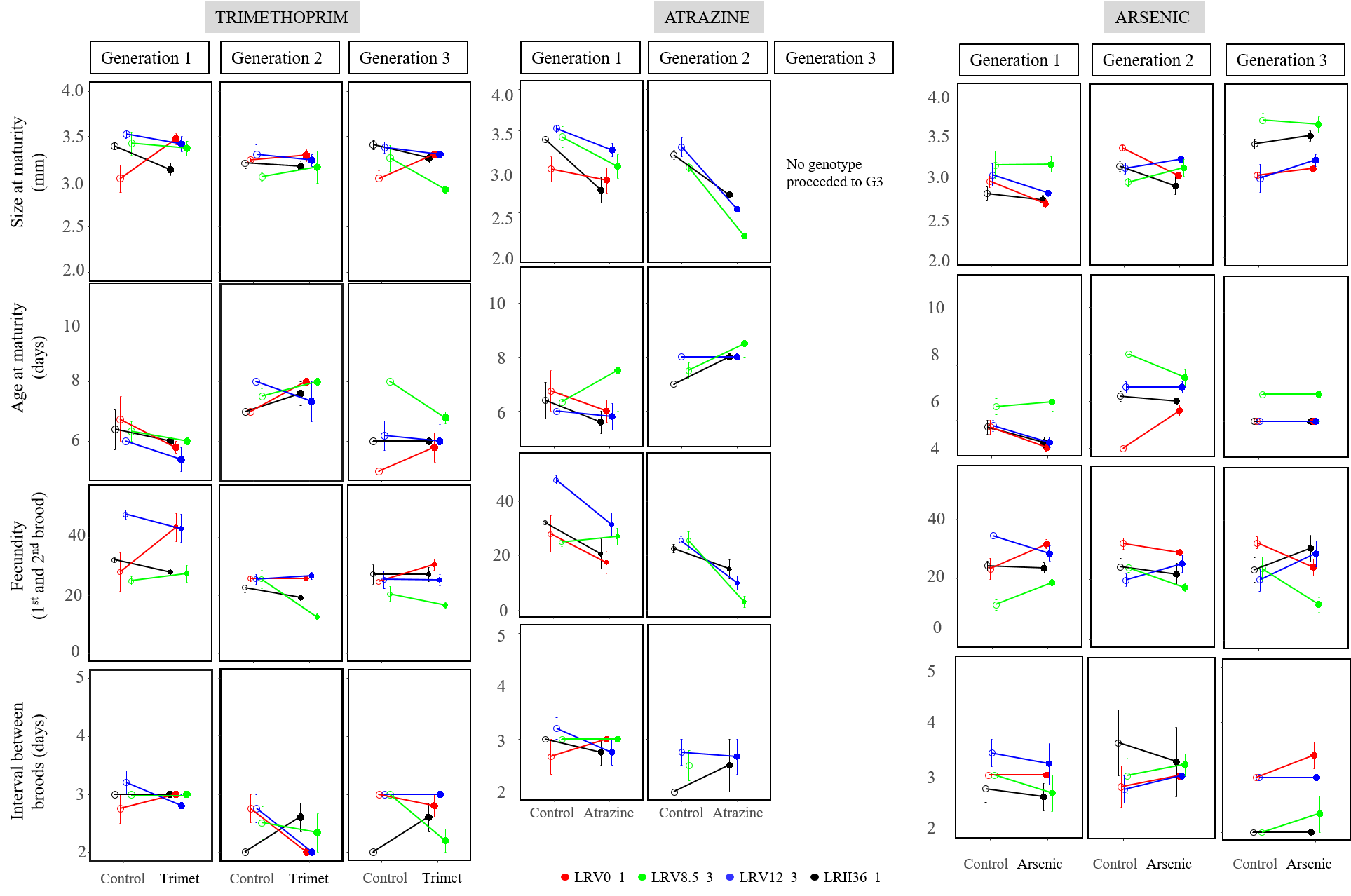


**Figure S2. Cross-generation survival plots.** Survival plots per genotype and generation for exposure to PFOS (70ng/L); diclofenac (2mg/L); trimethoprim (2 mg/L); atrazine (0.2 mg/L) and arsenic (1,000 µg/L). The plots are obtained with a survival model fit via the “psm” function in the “rms” R package V.3.3. A separate model was fitted to each treatment. Genotypes are color-coded as in Figure 1: LRII36_1 (<1950; black); LRV12_3 (1960–1970; blue); LRV8.5_3 (1975–1985; green) and LRV0_1 (> 1999; red).


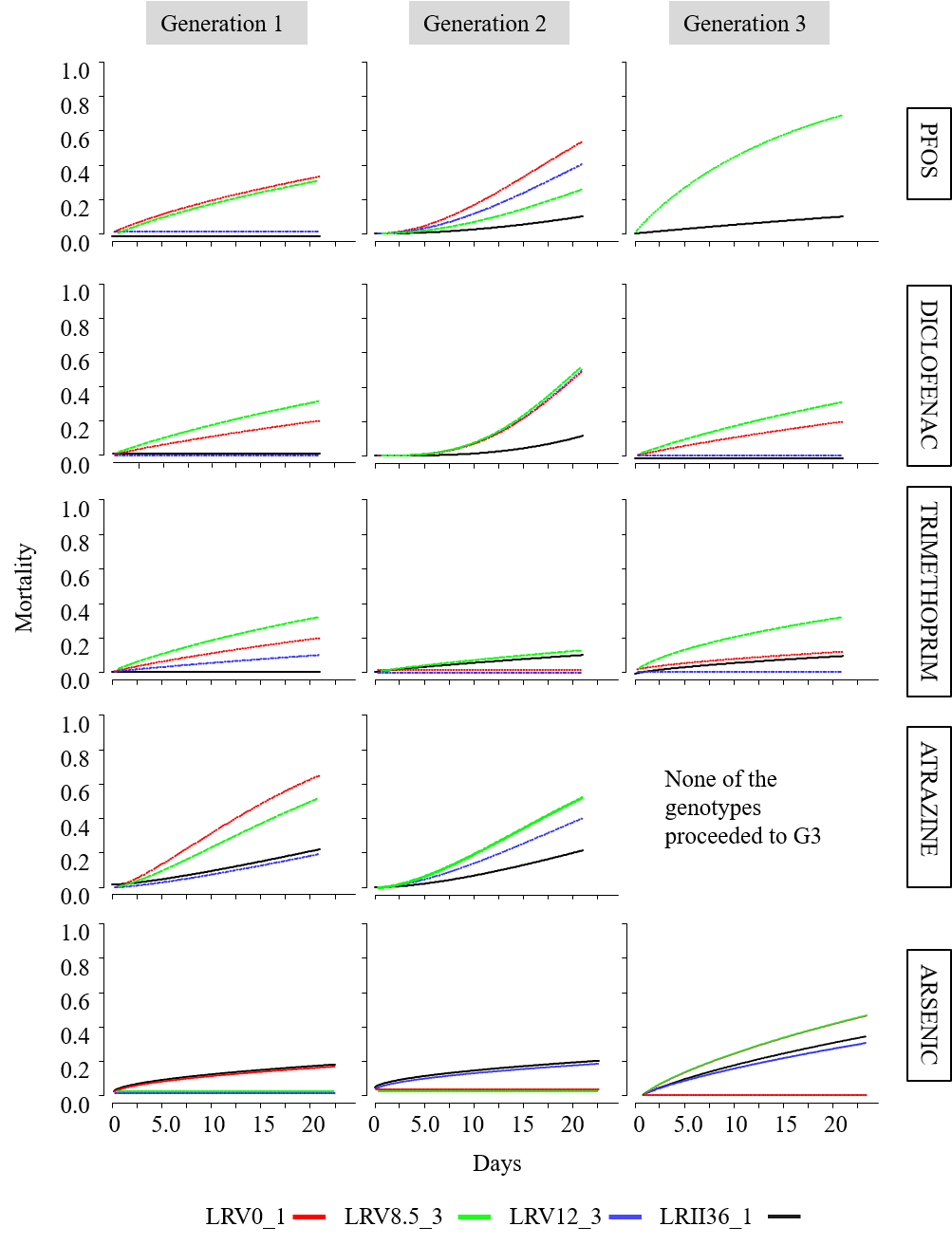

Supplement: Supplementary file 1 — Supplementary Material [file MEC-31-3098-s001.docx]
